# IgG4-assoziierte Orbitopathie als wichtige Differenzialdiagnose eines fortgeschrittenen Silent-Sinus-Syndroms

**DOI:** 10.1007/s00106-019-00798-9

**Published:** 2020-02-14

**Authors:** M. Jurkov, H. Olze, F. Klauschen, E. Bertelmann, U. Schneider, P. Arens

**Affiliations:** 1 HNO Klinik, Charité – Universitätsmedizin Berlin, Charitéplatz 1, 10117 Berlin, Deutschland; 2 Institut für Pathologie, Charité – Universitätsmedizin Berlin, Charitéplatz 1, 10117 Berlin, Deutschland; 3 Klinik für Augenheilkunde, Charité – Universitätsmedizin Berlin, Charitéplatz 1, 10117 Berlin, Deutschland; 4 Klinik für Rheumatologie, Charité – Universitätsmedizin Berlin, Charitéplatz 1, 10117 Berlin, Deutschland

**Keywords:** IgG4-related disease, Immunoglobulin G, Orbit, Eosinophilic angiocentric fibrosis, Chronic sinusitis, IgG4-assoziierte Erkrankung, Immunglobulin G, Orbita, Eosinophile angiozentrische Fibrose, Chronische Sinusitis

## Abstract

**Hintergrund:**

Mit Immunglobulin (Ig)G4 assoziierte Erkrankungen werden als immunvermittelte Erkrankungen klassifiziert. Die Ätiologie dieser Krankheiten ist bisher noch nicht geklärt. Sie manifestieren sich auf verschiedene Weise, und die gleichzeitige Beteiligung mehrerer Organe ist nicht ungewöhnlich.

**Kasuistik:**

Es wird der Fall eines Patienten vorgestellt, der in die Klinik der Autoren überwiesen wurde, nachdem mehrere erfolglose Nasennebenhöhlenoperationen bei ihm durchgeführt worden waren; bei Vorliegen eines Enophthalmus und einer resultierenden Durchwanderungskeratitis bestand die Verdachtsdiagnose eines Silent-Sinus-Syndroms. Der Erhalt der Orbita war nicht mehr möglich. Nach 5 Jahren ohne definitive Diagnose wurde nun die Diagnose einer IgG4-assoziierten Erkrankung gesichert.

**Diskussion:**

IgG4-assoziierte Erkrankungen stellen einen wichtigen Baustein bei der Differenzialdiagnose chronischer fortgeschrittener Erkrankungen der Orbita und der Nasennebenhöhlen dar. Bei unklaren Krankheitszeichen sollte diese Diagnose in Erwägung gezogen werden. Zu den typischen histologischen Befunden gehören ein storiformes Muster der Fibrose, Vaskulopathie und Gewebeinfiltration durch IgG4-Plasmazellen.

## Abstract (en)

**Background:**

Immunoglobulin (Ig)G4-related disease is classified as an immune-mediated disease. The etiology of this condition has not been explained to date. Manifestations of the disease are diverse, and simultaneous involvement of multiple organs is not unusual.

**Case report:**

We report the case of a patient referred to us after multiple unsuccessful paranasal sinus operations who presented with enophthalmos and a resultant migratory keratitis with a suspected diagnosis of silent sinus syndrome. Preservation of the orbit was no longer feasible. After five years without a definitive diagnosis, we ascertained that this was a case of IgG4-related disease.

**Discussion:**

IgG4-related disease represents an important element in the differential diagnosis of chronic advanced diseases of the orbit and paranasal sinuses. The diagnosis should be considered in the case of unclear disease presentations. Typical histological findings include a storiform pattern of fibrosis, vasculopathy, and tissue infiltration by IgG4 plasma cells.

## Hintergrund

IgG4-assoziierte Erkrankungen sind eine Gruppe von immunologisch vermittelten Erkrankungen, die verschiedene Organe betreffen können. Betroffen sein können typischerweise die Orbita, die Speicheldrüsen, die Lunge, die Bauchspeicheldrüse, die Gallengänge und das retroperitoneale Gewebe [1, 2]. Die Kopf-Hals-Region ist am zweithäufigsten nach dem Pankreas betroffen [3]. Bei einer Orbitabeteiligung werden am häufigsten die Tränendrüsen und die extraokulären Muskeln in Mitleidenschaft gezogen. Andere Teile der Orbita sowie benachbarte Strukturen können aber ebenfalls betroffen sein. Ein wesentliches Merkmal ist die orbitale Schwellung bzw. Proptosis [4]. Eine Mitbeteiligung der Nase und der Nasennebenhöhle kann vorkommen. Auch isolierte Manifestationen in diesem Bereich sind beschrieben worden [5].

## Fallbericht

Es wird über einen 77 Jahre alten männlichen Patienten berichtet, der sich mit der Verdachtsdiagnose eines Silent-Sinus-Syndroms links in der Ambulanz der Autoren vorstellte. Er litt unter zunehmendem Visusverlust, Doppelbildern und Enophthalmus. In der Untersuchung mittels sphärischem Korrekturglas von −2 Dioptrien wurde ein Visus von 0,125 ermittelt. Es zeigte sich eine Okkulomotoriusparese. Im Hals-Nasen-Ohren-ärztlichen Untersuchungsbefund war links keine mediale Kieferhöhlenwand mehr abgrenzbar. Der Patient befand sich in gutem Allgemein- und Ernährungszustand. Nebenerkrankungen waren ein stattgehabter Apoplex, koronare Herzerkrankung, Diabetes mellitus Typ 2 sowie eine chronische Niereninsuffizienz.

**Figure 1:**
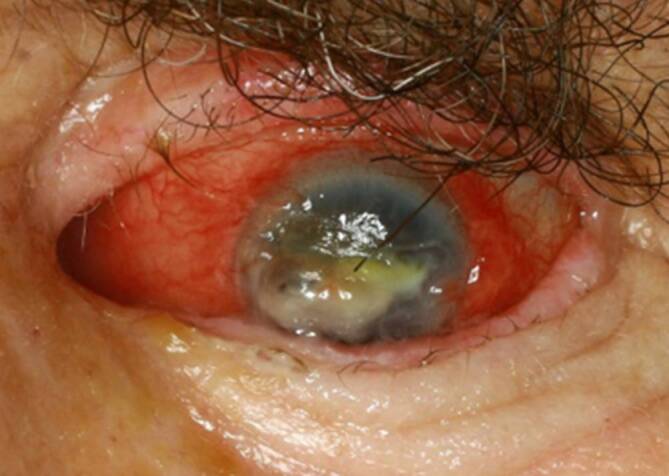

Der Patient gab an, dass die Symptomatik seit 5 Jahren bestehe. Seit einem Jahr sei er auf dem betroffenen Auge nahezu blind. Er gab an, unter der Verdachtsdiagnose eines Silent-Sinus-Syndroms bereits an den Nasennebenhöhlen operiert worden zu sein. Ein kurzfristiger Wiedervorstellungstermin zur weiteren Diagnostik hat der Patient aufgrund eines Oberschenkelhalsbruchs und anschließender Rehabilitation nicht wahrgenommen. Der Patient stellte sich erst 2 Monate später wieder in der Klinik der Autoren vor. Zu diesem Zeitpunkt präsentierte sich der Patient mit einer fortgeschrittenen Durchwanderungskeratitis. Es bestand Ptosis, Einschränkung der Bulbusmotilität und Blindheit auf der betroffenen Seite (Abb. 1).

**Figure 2:**
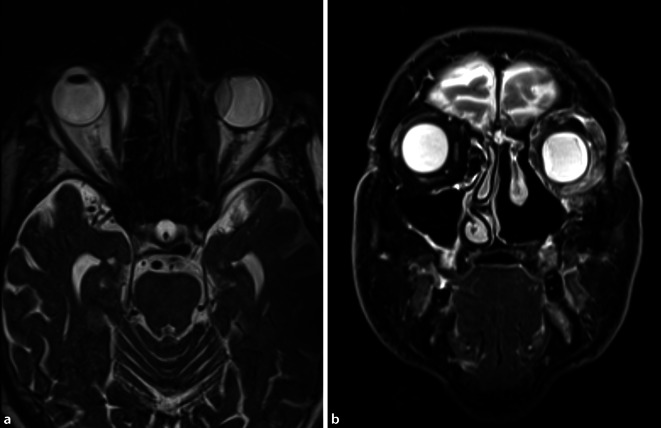

Die Magnetresonanztomographie des Mittelgesichts ergab das Bild einer chronischen Sinusitis bei mutmaßlich radikal endonasal voroperierten Nasennebenhöhlen. Intraorbital zeigte sich ein kontrastmittelaufnehmendes Enhancement bis in den Orbitatrichter und über die Fissura orbitalis superior bis nach intrakraniell reichend ohne Hinweise für eine zerebrale Beteiligung (Abb. 2).

**Figure 3:**
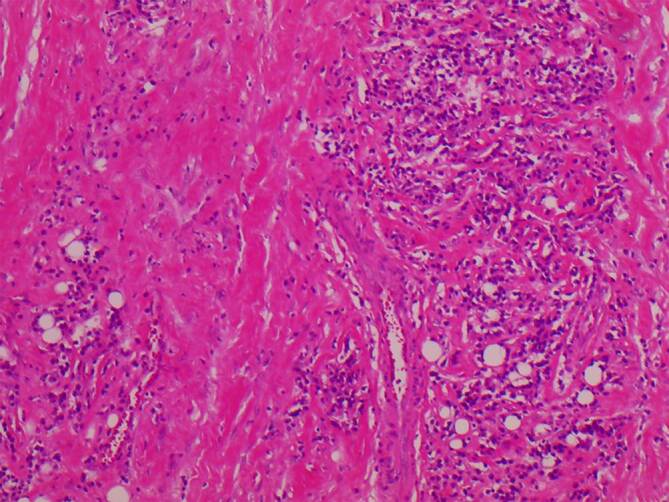

Aufgrund der fortgeschrittenen Durchwanderungskeratitis mit bakterieller Superinfektion bei unklarer Krankheitsursache und funktionslosem Auge wurde der interdisziplinäre Entscheid zur diagnostischen und therapeutischen Exenteratio orbitae links getroffen. Bei zunehmender Destruktion des Nasennebenhöhlensystems links wurde im gleichen Eingriff zudem eine transorbitale Nasennebenhöhlensanierung durchgeführt. Der operative Eingriff verlief komplikationslos. Im weiteren Verlauf erlitt der Patient jedoch einen Herzinfarkt sowie ein postinterventionelles Delir. Nach intensivmedizinischer Behandlung und anschließender stationären Überwachung konnte der Patient am 44. postoperativen Tag entlassen werden. In der histopathologischen Aufarbeitung des Enukleats fand sich eine chronisch-fibrosierende Entzündung mit einem erhöhten Gehalt an IgG4-positiven Plasmazellen (Abb. 3 und 4). Es wurde daraufhin der Verdacht auf eine IgG4-assoziierte Erkrankung geäußert. Im Serum des Patienten wurde ein erhöhter IgG1-Titer (8,820 g/l, Referenzwert: 2,8–8 g/l) und ein erhöhter IgG4-Titer (1,659 g/l, Referenzwert: 0,052–1,250g/l) nachgewiesen. In Zusammenschau der Befunde wurde die Diagnose einer IgG4-assoziierten Orbitopathie mit Beteiligung der Nasennebenhöhlen gestellt. Aufgrund der nun als ausreichen erachteten Sanierung des Befundes mit gutem Verlauf der Wundheilung wurde vonseiten der Kollegen der Klinik für Rheumatologie eine „Watch-and-wait-Strategie“ mit dem Patienten vereinbart. Der Patient starb 15 Monate postoperativ an einem Herzinfarkt.

**Figure 4:**
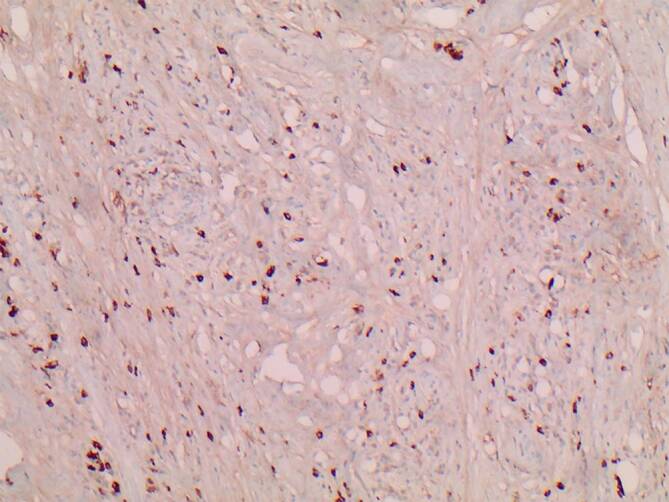

## Diskussion

Zu den Hauptmerkmalen der Manifestation einer IgG4-assoziierten Erkrankung gehört die tumorähnliche Schwellung der betroffenen Organe, die mit der Infiltration des lymphatischen Gewebes durch IgG4-positive Plasmazellen, „storiformer“ Fibrose und mit obliterativer Venenentzündung einhergeht [6, 7]. Die Anzahl der IgG4-positiven Plasmazellen pro Hochleistungsfeld (HPF), die als direktes diagnostisches Korrelat gilt, variiert von Gewebe zu Gewebe. Im Allgemeinen beträgt das Minimum für die Diagnosestellung 30–50 IgG4-positive Zellen/HPF. In der Tränendrüse können jedoch auch 10 IgG4-positive Plasmazellen/HPF für die Diagnose ausreichend sein [8, 9]. Storiforme Fibrose und obliterative Phlebitis sind eher typisch für die Pathologie. Jedoch sind sie nicht immer bei der Orbitabeteiligung vorhanden [7–9]. Bei Patienten mit typischen klinischen Zeichen und Organbeteiligungen werden sowohl die Messung des Serum-IgG4-Spiegels als auch die Gewebebiopsie empfohlen [6, 10]. Erhöhte Serumkonzentrationen von IgG4 finden sich bei 60–70 % der Patienten. Der Nachweis von IgG4 im Serum ist jedoch unspezifisch, da er auch mit dem Churg-Strauss-Syndrom, Sarkoidose und allergischen Erkrankungen in Verbindung gebracht werden kann [11].

Zusammenfassend gibt es 3 diagnostische Hauptkriterien: diffuse oder fokale Beteiligung eines oder mehrerer Organe, erhöhter IgG4-Serumspiegel, typische Histologie, bestehend aus dem dichten lymphoplasmazellulären Entzündungsinfiltrat, u. a. aus IgG4-positiven Plasmazellen, und mit gefäßassoziierter Entzündung im Sinne einer obliterativen, lumenverlegenden Phlebitis [1, 7]. Für die Induktion einer Remission stellen Glukokortikoide das Mittel der ersten Wahl dar. Der Induktionstherapie folgt meist eine Erhaltungstherapie. Die meisten Patienten sprechen innerhalb von einigen Wochen auf Glukokortikoide an, typischerweise mit symptomatischer Besserung, Verkleinerung der vergrößerten Organe, Verbesserung der Organfunktion und einer Reduktion des IgG4-Serumspiegels. Das Therapiemittel der zweiten Wahl stellt Rituximab dar [12, 13].

Der hier beschriebene Fall ist aufgrund mehrerer Faktoren besonders. Es handelt sich um einen gleichzeitigen Befall der paranasalen Sinus und der Orbita. Es existieren wenige Fallberichte, die eine ähnliche, jedoch nicht identische Befundkonstellation beschreiben [14, 15]. Inwieweit ältere Fallberichte zur eosinophilen angiozentrischen Fibrose den IgG4-assoziierten Erkrankungen der Nase und der Orbita zugeordnet werden müssten, ist im einzelnen Fall unklar. Eine retrospektive Auswertung von Deshpande et al. legt diesen Schluss jedoch nahe [16]. Zudem illustriert dieser Fall eindrucksvoll den Verlauf der Erkrankung bei 5‑jähriger unzureichender Therapie. Unglücklicherweise war es den Autoren nicht möglich, die auswärtigen histopathologischen Befunde zur Nachuntersuchung anzufordern, sodass eine komplette Rekonstruktion des Krankheitsverlaufs nicht gelang.

Schlussendlich zeigt dieser Fall, dass die orbitale Beteiligung einer IgG4-assoziierten Erkrankung nicht zwingend mit einer Proptosis einhergehen muss, sondern bei Destruktion der angrenzenden knöchernen Strukturen und Sinus ebenso mit einem Enophthalmus einhergehen kann.

## Fazit für die Praxis

- IgG4-assoziierte Erkrankungen können die Nasennebenhöhlen und gleichzeitig die Orbita befallen.
- Nicht immer geht dies mit der häufig beschriebenen Proptosis einher, sodass dieses Krankheitsbild differenzialdiagnostisch auch bei destruierenden Prozessen mit einhergehendem Enophthalmus in Betracht gezogen werden sollte.
